# Pretreatment C-Reactive Protein/Albumin Ratio is Associated With Poor Survival in Patients With 2018 FIGO Stage IB-IIA HPV-Positive Cervical Cancer

**DOI:** 10.3389/pore.2021.1609946

**Published:** 2021-12-21

**Authors:** Yinan Jiang, Haifeng Gu, Xiaojing Zheng, Baoyue Pan, Pingping Liu, Min Zheng

**Affiliations:** Department of Gynecology, State Key Laboratory of Oncology in South China, Collaborative Innovation Center for Cancer Medicine, Sun Yat-Sen University Cancer Center, Guangzhou, China

**Keywords:** recurrence, survival, CAR, prognostic markers, UCC, HPV

## Abstract

**Objectives:** The present study aimed to identify the predictive value of inflammatory indexes stratified according to human papillomavirus (HPV) infection status in women with FIGO 2018 stage IB∼IIA cervical cancer. We also explored the influences of HPV infection status on the survival of cervical cancer patients.

**Methods:** We collected data for 583 women with stage IB∼IIA cervical cancer in Sun Yat-sen University Cancer Center between 2009 and 2017. The *t*-test, chi-squared (χ^2^) test and Fisher’s exact test were applied to compare the differences of inflammatory indexes and clinicopathological features between HPV-positive and HPV-negative groups. Univariate and multivariate analyses were used to identify clinicopathological factors that were associated with the prognosis of cervical cancer patients.

**Results:** There were no differences in overall survival (OS) and progression-free survival (PFS) between HPV-positive and HPV-negative groups. In HPV-positive group, the maximum tumor size, neoadjuvant chemotherapy and the body mass index (BMI) correlated significantly with C-reactive protein/albumin ratio (CAR). The maximum tumor size and the prognostic nutritional index (PNI) correlated significantly with the platelet-lymphocyte ratio (PLR). The maximum tumor size, neoadjuvant chemotherapy and PLR correlated significantly with PNI. Univariate and multivariate analyses showed that the depth of tumor invasion (HR: 3.651, 95% CI: 1.464–9.103, *p* = 0.005; HR: 2.478, 95% CI: 1.218–5.043, *p* = 0.012) and CAR (HR: 5.201, 95% CI: 2.080–13.004, *p* < 0.0001; HR: 2.769, 95% CI: 1.406–5.455, *p* = 0.003) were independent predictors of poor OS and PFS. PNI was an independent protective factor of OS (HR: 0.341, 95% CI: 0.156–0.745, *p* = 0.007). PLR was an independent factor of PFS (HR: 1.991, 95% CI: 1.018–3.894, *p* = 0.044). In HPV-negative group, BMI correlated significantly with CAR. Only depth of invasion (HR: 9.192, 95% CI: 1.016–83.173, *p* = 0.048) was the independent predictor of poor OS, and no inflammation indexes were independent predictors of prognosis.

**Conclusion:** In patients with HPV-positive cervical cancer, depth of invasion, PNI and CAR are independent factors of OS, and depth of invasion, PLR and CAR are independent factors for PFS. For patients with HPV-negative disease, no inflammation indexes had predictive value for prognosis. The predictive value of inflammation indexes on prognosis is more significant in patients with HPV-positive cervical cancer. Stratification of HPV infection status promotes a more precise clinical application of inflammation indexes, thus improving their accuracy and feasibility.

## Introduction

Globally, uterine cervical cancer (UCC) still ranks fourth for both incidence and mortality among cancers, with about 570,000 new cases and 311,000 deaths each year [1]. To the best of our knowledge, high-risk human papillomavirus (HPV) infection is one of the main risk factors for cervical cancer, and the body inflammatory state influenced by HPV infection is related closely to the progression of cervical cancer [2]. With the popularization of cervical cancer screening and the widespread use of anti-HPV vaccines, the incidence of cervical cancer in developed countries has declined gradually [1–3]. However, because of a large population, uneven economic development, and late application of anti-HPV vaccines, the incidence of cervical cancer in China ranks second in the world, and the proportion of young patients is rising rapidly. Thus, predicting these patients’ life expectancy is vital for clinicians and patients [4]. Pelvic lymph node metastasis is acknowledged as one of the most important prognostic factors [5]. With the update of the Federation of Gynecologists and Obstetricians (FIGO) guidelines, cervical cancer patients with pelvic or para-aortic lymph node involvement are classified as stage IIIC [5, 6]. Therefore, for patients with stage IB∼IIA cervical cancer in the revised staging, the predictive factors that can be used preoperatively remain limited.

Indexes of a systemic inflammatory response (SIR), such as the C-reactive protein (CRP)-albumin (ALB) ratio (CAR), the platelet-lymphocyte ratio (PLR), the prognostic nutritional index (PNI), the neutrophil-lymphocyte ratio (NLR), and the lymphocyte-monocyte ratio (LMR), have been used not only to determine the nutritional status and complications but also to predict prognosis of patients in various cancers [7–11]. As a novel inflammation- and nutrition-based score, CAR has been reported as an independent indicator of poor prognosis in different cancers including lung cancer, colorectal cancer, pancreatic cancer and hepatocellular cancer [12–15]. Platelets are an important component of peripheral blood cells and have complex relations with tumor cells. It’s found that several proinflammatory mediators (e.g., IL1 and IL6) could stimulate and increase plateles counts. Therefore, platelet counts can reflect the inflammatory status to some extent [16]. Besides, platelets activate signalling pathways to induce epithelial-to-mesenchymal-like transition (EMT) and tumor metastasis [17]. Increasing evidences have proven that NLR is a reliable indicator of prognosis in solid tumors. In Zhang’s study with 460 Chinese cervical cancer patients treated with radical surgery, the results showed that there was a significant value of NLR for patients’ prognosis [18]. PLR is also demonstrated as an independent factor for worse OS in ovarian cancer and pancreatic cancer [16, 19]. PNI, which is calculated according to ALB levels and peripheral lymphocyte numbers, reflects both the nutritional and immunal status of patient. Several studies have demonstrated that PNI is a preditive indicator of prognosis in hepatocellular cancer, gastric cancer and pancreatic cancer [20–22]. In recent years, these indexes have gradually attracted attentions for their application as prognostic predictors in solid tumors [23–25].

Although several studies on survival and recurrence outcomes of patients with cervical cancer in various stages have been published, to date, there is no report concerning the impact of HPV infection status on the inflammatory indexes and the prediction of prognosis in patients with cervical cancer. In this study, we firstly divided the patients into HPV-positive group and HPV-negative group, and compared the clinical parameters and prognosis between two groups. We analyzed retrospectively the predictive value of preoperative inflammation markers (PLR, PNI, and CAR) on the prognosis of patients with FIGO 2018 stage IB∼IIA cervical cancer with different HPV infection statuses.

## Materials and Methods

### Ethics Statement

This retrospective study was performed in accordance with the Declaration of Helsinki and approved by the Ethics Committee of the Sun Yat-sen University Cancer Center Institutional Review Board (IRB-number B2020-345-01). Informed patient consent was waived in this observational and noninterventional study, in which the patient’s data were kept under strict control. The authenticity of this article has been validated by uploading the key raw data onto the Research Data Deposit public platform (www.researchdata.org.cn), with the approval RDD number as RDDA2021001939.

### Patients

In this study, a total of 583 patients with FIGO stage IB∼IIA cervical cancer who had received radical resection at Sun Yat-sen University Cancer Center between February 2009 and December 2017 were enrolled as a cohort. Every patient’s postoperative histopathological type was reviewed according to the WHO criteria by two experienced gynecological pathologists. Routine blood examinations were performed before surgery. The study excluded patients who had hematological malignancies, other malignancies, autoimmune diseases, active infection, or inflammatory disease within the month before blood examinations, or who lost contact. The tumor stage of all cases was defined according to the FIGO 2018 cervical cancer criteria [6].

### Data Collection

Clinical data, including clinicopathological parameters (such as age at diagnosis, height, weight, histopathological type, tumor grade, maximal tumor size, depth of invasion, preoperative neoadjuvant chemotherapy, postoperative adjuvant therapy, FIGO stage, and HPV infection status), survival time, and preoperative routine blood examination results (including absolute counts of leukocytes, lymphocytes and platelets, the levels of CRP and ALB) were collected retrospectively from the medical records. In addition, we also collected the postoperative adjuvant treatment including radiotherapy and chemotherapy.

### Parameters Calculations

Venous blood was drawn from all patients within 1 week before surgery. The CAR, PLR, PNI and body mass index (BMI) were calculated using the following equations:

$$\text{CAR}=\frac{\text{CRP}\left(\text{mg}/\text{L}\right)}{\text{ALB}\left(\text{g}/\text{L}\right)}$$

$$\text{PLR}=\frac{\text{platelets}\;\text{counts}\left(10^{9}/\text{L}\right)}{\text{lymphocytes}\;\text{counts}\left(10^{9}/\text{L}\right)}$$

$$\text{PNI}=\text{ALB}\left(\text{g}/\text{L}\right)+5*\text{lymphocytes}\;\text{counts}\left(10^{9}/\text{L}\right)$$

$$\text{BMI}=\frac{\text{weight}\left(\text{kg}\right)}{{\text{height}}^{2}²\left({\text{m}}^{2}²\right)}$$

$$\mathrm{The}\;\mathrm{depth}\;\mathrm{of}\;\mathrm{invasion}=\frac{\mathrm{tumor}\;\mathrm{invasion}\;\mathrm{in}\;\mathrm{mm}}{\mathrm{cervical}\;\mathrm{wall}\;\mathrm{thickness}\;\mathrm{in}\;\mathrm{mm}}$$

The depth of invasion was evaluated by two experienced gynecological pathologists. The invasion of outer one-third (≥2/3) of the cervical stroma was considered as a risk factor that increases the risk of pelvic recurrence [6].

Receiver operating characteristic (ROC) curves were generated for pre-treatment CAR, PLR, and PNI to determine the cut-off values that yielded optimal sensitivity and specificity. The upper right point of the ROC curve was selected, and the Youden index was calculated according to the sensitivity and specificity of each possible point in the statistical results. Then, the largest point was selected to determine the optimal cut-off value of the hematological index, such that the numerical variable could be transformed into a classified variable to analyze the statistical data.

### HPV Testing

Every patient accepted high-risk type HPV test before surgery. Cervical cells sample was collected by the Digene Hybrid Capture 2 (HC2) HPV DNA Collection Device (QIAGEN, Germantown, MD, United States), which consisted of a cervical brush and a digene specimen transport medium. The DNA copy number of the high-risk type HPV was calculated. If the copy number was 0–1 pg/ml, it indicated that there was no HPV infection. When the copy number was greater than 1 pg/ml, it indicated a positive HPV infection.

### Neoadjuvant and Adjuvant Therapy

Preoperative neoadjuvant chemotherapy was administered to inhibit some micrometastasis and distant metastasis and to improve the radical curability and safety of surgery. Patients whose tumor could not be removed completely in the beginning were given 2 cycles of cisplatin (70 mg/m^2^) and paclitaxel (175 mg/m^2^), every 3 weeks before surgery.

According to the FIGO 2018 and NCCN guidelines, patients with either positive surgical margins or lymph node metastases or parametrial spread, were offered postoperative radiotherapy with chemotherapy. For adenocarcinoma and adenosquamous carcinoma patients with two of four risk factors (deep stromal invasion, tumor size more than 3 cm, lymphovascular invasion, adenocarcinoma), and for squamous cell carcinoma patients with two of three risk factors (deep stromal invasion, tumor size more than 4 cm, lymphovascular invasion), postoperative radiotherapy was required. Among other patients, those with poorly differentiated grade and tumor size more than 2 cm, or highly maligant pathological type such as clear cell adenocarcinoma, neuroendocrine carcinoma, were given chemotherapy without radiotherapy. All other patients following radical hysterectomy did not need any adjuvant therapy [6, 26].

### Follow-Up

After the initial treatment, follow-up assessments were performed once every 3 months for the first 2 years, once every 6 months for the next 3 years, and then once every year after 5 years. The main examinations included routine blood tests, tumor markers measurement, gynecological examinations, chest radiography, and computed tomography (CT) or magnetic resonance imaging (MRI) scans. In cases with abnormal findings and suspected tumor recurrence, lesion biopsy was performed to determine whether recurrence had occurred. As study endpoints, OS was defined as duration from the date of initial surgery to the date of death for any causes or the last follow-up (December 31st, 2020), and PFS was defined as the time interval from the date of surgery to progression or relapse.

### Statistical Analysis

Comparison analyses of inflammatory indexes and clinicopathological variables between HPV-positive and HPV-negative groups were conducted using *t*-test, Pearson’s chi-squared (χ^2^) test and Fisher’s exact test. Comparison analyses of clinicopathological variables between high- and low-inflammatory index groups were performed as well.

The survival curves were plotted using the Kaplan–Meier method, and log-rank tests were carried out to assess survival differences between groups. Significant prognostic variables in the univariate analysis were selected for the multivariate Cox Regression model analysis to identify independent prognostic variables using the forward stepwise method. Analyses were performed using SPSS 23.0 software (IBM Corporation, Armonk, NY, United States) and GraphPad Prism 8 software (GraphPad Software, Inc., San Diego, CA, United States). A two sided *p* < 0.05 indicated that the difference was considered statistically significant.

## Results

### Patients’ Characteristics

In total, 583 patients with stage IB∼IIA cervical cancer were recruited in this study, of which 501 (85.93%) cases were HPV-positive and 82 (14.07%) cases were HPV-negative. The associations of characteristics between two groups were shown in Table 1. All patients had no lymph node metastasis. The median age at the time of prognosis was 48.88 ± 9.174 years, and the median follow-up time was 68.04 ± 26.32 months. The HPV-positive group had a higher proportion of squamous cell carcinoma type compared to HPV-negative group (χ^2^ = 35.962, *p* < 0.0001). The proportions of other characteristics were relatively balanced. During the follow-up period, a total of 42 patients experienced recurrence or progression, and 31 of them died.

**TABLE 1:** Comparison of clinical characteristics between patients with HPV-positive and HPV-negative cervical cancer. The bold values mean that the difference is considered statistically significant (*p* < 0.05).

Variables	HPV-positive (*n* = 501)	HPV-negative (*n* = 82)	Statistics	*p*-Value
Median follow-up, months	68.34 ± 26.93	66.23 ± 22.29	*t* = 0.6720	0.5019
CAR	0.0642 ± 0.135	0.0932 ± 0.284	*t* = 1.483	0.1386
PLR	132.0 ± 50.75	131.0 ± 51.55	*t* = 0.1691	0.8658
PNI	53.47 ± 4.409	53.34 ± 4.681	*t* = 0.2464	0.8055
Age	49.05 ± 9.208	47.87 ± 8.954	χ^2^ = 0.131	0.717
≤45 years	173 (34.5)	30 (36.6)		
>45 years	328 (65.5)	52 (63.4)		
FIGO stage			χ^2^ = 3.393	0.065
IB	327 (65.3)	62 (75.6)		
IIA	174 (34.7)	20 (24.4)		
Maximum tumor size			χ^2^ = 1.004	0.316
≤4 cm	414 (82.6)	64 (78.0)		
>4 cm	87 (17.4)	18 (22.0)		
Pathological type			**χ** ^**2**^ **= 35.962**	**<0.0001**
Squamous	423 (84.4)	46 (56.1)		
Non-squamous	78 (15.6)	36 (43.9)		
Neoadjuvant chemotherapy			χ^2^ = 2.258	0.133
No	392 (78.2)	58 (70.7)		
Yes	109 (21.8)	24 (29.3)		
Histological grade			χ^2^ = 3.050	0.081
G1 + G2	217 (43.3)	44 (53.7)		
G3	284 (56.7)	38 (46.3)		
Depth of invasion			χ^2^ = 3.291	0.070
<2/3	270 (53.9)	53 (64.6)		
≥2/3	231 (46.1)	29 (35.4)		
BMI, kg/m^2^			χ^2^ = 0.010	0.921
<19 or >24	229 (45.7)	37 (45.1)		
19–24	272 (54.3)	45 (54.9)		

HPV, human papillomavirus; CAR, C-reactive protein/albumin ratio; PLR, platelet-lymphocyte ratio; PNI, prognostic nutritional index; FIGO, Federation of Gynecologists and Obstetricians; Depth of invasion, tumor invasion in mm/cervical wall thickness in mm; BMI, body mass index.

In this study, 109 HPV-positive cases and 24 HPV-negative cases received neoadjuvant chemotherapy before surgery. Patients with risk factors were required to accept radiotherapy or chemotherapy after surgery. In HPV-positive group, there were 151 cases without postoperative adjuvant therapy, 96 cases with chemotherapy, 111 cases with radiotherapy and 143 cases with chemotherapy and radiotherapy. In HPV-negative group, there were 28 cases without postoperative adjuvant therapy, 17 cases with chemotherapy, 12 cases with radiotherapy and 25 cases with chemotherapy and radiotherapy. We compared the prognosis of different subgroups and found that there was no influence on prognosis of cervical cancer patients no matter what kind of postoperative adjuvant therapy they received (Supplementary Figure S1).

### Cut-Off Values of Inflammatory Indexes

The cut-off values of CAR, PNI, and PLR were determined using ROC curves (Supplementary Figure S2). The analyses identified CAR ≤ 0.0396 (area under the curve (AUC): 0.619, 66.7% sensitivity, 61.9% specificity), PLR ≤ 163.41 (AUC: 0.509, 33.3% sensitivity, 78.9% specificity), PNI ≤ 50.15 (AUC: 0.520, 66.7% sensitivity, 21.3% specificity), as the most accurate cut-off values for patients with cervical cancer.

### Comparison Between Inflammatory Indexes and Clinicopathological Variables

Patients with different HPV infection statuses were divided into two groups according to the cut-off values of CAR, PNI, or PLR. The associations of clinical characteristics between CAR^hi^ and CAR^lo^, PLR^hi^ and PLR^lo^, PNI^hi^ and PNI^lo^ groups were analyzed using *t*-test, chi-squared (χ^2^) test and Fisher’s exact test in HPV-positive group and HPV-negative group, respectively.

We found that although there were no differences in age, FIGO stage, and pathological type among patients with HPV-positive cervical cancer. A higher CAR level (>0.0396) correlated significantly with larger tumor size (χ^2^ = 8.360, *p* = 0.004), higher receipt rate of neoadjuvant chemotherapy (χ^2^ = 6.350, *p* = 0.012) and nonstandard BMI (χ^2^ = 16.962, *p* < 0.0001) (Table 2). A higher level of PLR (>163.41) was associated with larger tumor size (χ^2^ = 4.086, *p* = 0.043) and lower PNI (χ^2^ = 49.013, *p* < 0.0001) (Table 3). While a higher level of PNI (>50.15) was associated with smaller tumor size (χ^2^ = 6.139, *p* = 0.013), lower receipt rate of neoadjuvant chemotherapy (χ^2^ = 4.190, *p* = 0.041) and lower PLR (χ^2^ = 49.013, *p* < 0.0001) (Table 4).

**TABLE 2:** Comparison of clinical characteristics between preoperative CAR^lo^ and CAR^hi^ groups in patients with HPV-positive cervical cancer. The bold values mean that the difference is considered statistically significant (*p* < 0.05).

Variables	Case (*n* = 501)	CAR ≤ 0.0396 (*n* = 305)	CAR > 0.0396 (*n* = 196)	χ^2^	*p*-Value
Age				1.196	0.274
≤45 years	173	111 (36.4)	62 (31.6)		
>45 years	328	194 (63.6)	134 (68.4)		
FIGO stage				0.042	0.837
IB	327	198 (64.9)	129 (65.8)		
IIA	174	107 (35.1)	67 (34.2)		
Maximum tumor size				8.360	**0.004**
≤4 cm	414	264 (86.6)	150 (76.5)		
>4 cm	87	41 (13.4)	46 (23.5)		
Pathological type				1.939	0.164
Squamous	423	252 (82.6)	171 (87.2)		
Non-squamous	78	53 (17.4)	25 (12.8)		
Neoadjuvant chemotherapy				6.350	**0.012**
No	392	250 (82.0)	142 (72.4)		
Yes	109	55 (18.0)	54 (27.6)		
Histological grade				0.027	0.869
G1 + G2	217	133 (43.6)	84 (42.9)		
G3	284	172 (56.4)	112 (57.1)		
Depth of invasion				0.444	0.505
<2/3	270	168 (55.1)	102 (52.0)		
≥2/3	231	137 (44.9)	94 (48.0)		
PLR				0.555	0.456
≤163.41	392	242 (79.3)	150 (76.5)		
>163.41	109	63 (20.7)	46 (23.5)		
PNI				1.510	0.219
≤50.15	111	62 (20.3)	49 (25.0)		
>50.15	390	243 (79.7)	147 (75.0)		
BMI, kg/m^2^				16.962	**<0.0001**
<19 or >24	229	117 (38.4)	112 (57.1)		
19–24	272	188 (61.6)	84 (42.9)		

FIGO, Federation of Gynecologists and Obstetricians; Depth of invasion, tumor invasion in mm/cervical wall thickness in mm; CAR, C-reactive protein/albumin ratio; PLR, platelet-lymphocyte ratio; PNI, prognostic nutritional index; BMI, body mass index.

**TABLE 3:** Comparison of clinical characteristics between preoperative PLR^lo^ and PLR^hi^ groups in patients with HPV-positive cervical cancer. The bold values mean that the difference is considered statistically significant (*p* < 0.05).

Variables	Case (*n* = 501)	PLR ≤ 163.41 (*n* = 392)	PLR > 163.41 (*n* = 109)	χ^2^	*p*-Value
Age				0.007	0.934
≤45 years	173	135 (34.4)	38 (34.9)		
>45 years	328	257 (65.6)	71 (65.1)		
FIGO stage				0.238	0.626
IB	327	258 (65.8)	69 (63.3)		
IIA	174	134 (34.2)	40 (36.7)		
Maximum tumor size				4.086	**0.043**
≤4 cm	414	331 (84.4)	83 (76.1)		
>4 cm	87	61 (15.6)	26 (23.9)		
Pathological type				0.819	0.366
Squamous	423	334 (85.2)	89 (81.7)		
Non-squamous	78	58 (14.8)	20 (18.3)		
Neoadjuvant chemotherapy				1.265	0.261
No	392	311 (79.3)	81 (74.3)		
Yes	109	81 (20.7)	28 (25.7)		
Histological grade				0.153	0.696
G1 + G2	217	168 (42.9)	49 (45.0)		
G3	284	224 (57.1)	60 (55.0)		
Depth of invasion				1.061	0.303
<2/3	270	216 (55.1)	54 (49.5)		
≥2/3	231	176 (44.9)	55 (50.5)		
CAR				0.555	0.456
≤0.0396	305	242 (61.7)	63 (57.8)		
>0.0396	196	150 (38.3)	46 (42.2)		
PNI				49.013	**<0.0001**
≤50.15	111	60 (15.3)	51 (46.8)		
>50.15	390	332 (84.7)	58 (53.2)		
BMI, kg/m^2^				0.032	0.858
<19 or >24	229	180 (45.9)	49 (45.0)		
19–24	272	212 (54.1)	60 (55.0)		

FIGO, Federation of Gynecologists and Obstetricians; Depth of invasion, tumor invasion in mm/cervical wall thickness in mm; CAR, C-reactive protein/albumin ratio; PLR, platelet-lymphocyte ratio; PNI, prognostic nutritional index; BMI, body mass index.

**TABLE 4:** Comparison of clinical characteristics between preoperative PNI^lo^ and PNI^hi^ groups in patients with HPV-positive cervical cancer. The bold values mean that the difference is considered statistically significant (*p* < 0.05).

Variables	Case (*n* = 501)	PNI≤50.15 (*n* = 111)	PNI>50.15 (*n* = 390)	χ^2^	*p*-value
Age				0.023	0.879
≤45 years	173	39 (35.1)	134 (34.4)		
>45 years	328	72 (64.9)	256 (65.6)		
FIGO stage				0.015	0.901
IB	327	73 (65.8)	254 (65.1)		
IIA	174	38 (34.2)	136 (34.9)		
Maximum tumor size				6.139	**0.013**
≤4 cm	414	83 (74.8)	331 (84.9)		
>4 cm	87	28 (25.2)	59 (15.1)		
Pathological type				0.045	0.831
Squamous	423	93 (83.8)	330 (84.6)		
Non-squamous	78	18 (16.2)	60 (15.4)		
Neoadjuvant chemotherapy				4.190	**0.041**
No	392	79 (71.2)	313 (80.3)		
Yes	109	32 (28.8)	77 (19.7)		
Histological grade				1.653	0.199
G1 + G2	217	54 (48.6)	163 (41.8)		
G3	284	57 (51.4)	227 (58.2)		
Depth of invasion				0.031	0.859
<2/3	270	59 (53.2)	211 (54.1)		
≥2/3	231	52 (46.8)	179 (45.9)		
CAR				1.510	0.219
≤0.0396	305	62 (55.9)	243 (62.3)		
>0.0396	196	49 (44.1)	147 (37.7)		
PLR				49.013	**<0.0001**
≤163.41	392	60 (54.1)	332 (85.1)		
>163.41	109	51 (45.9)	58 (14.9)		
BMI, kg/m^2^				0.141	0.708
<19 or >24	229	49 (44.1)	180 (46.2)		
19–24	272	62 (55.9)	210 (53.8)		

FIGO, Federation of Gynecologists and Obstetricians; Depth of invasion, tumor invasion in mm/cervical wall thickness in mm; CAR, C-reactive protein/albumin ratio; PLR, platelet-lymphocyte ratio; PNI, prognostic nutritional index; BMI, body mass index.

For HPV-negative group, the results showed that a higher CAR level was related to nonstandard BMI (χ^2^ = 6.786, *p* = 0.009) (Table 5), and there was no difference of clinicopathological variables between PLR^hi^ and PLR^lo^ groups (Table 6) as well as PNI^hi^ and PNI^lo^ groups (Table 7).

**TABLE 5:** Comparison of clinical characteristics between preoperative CAR^lo^ and CAR^hi^ groups in patients with HPV-negative cervical cancer. The bold values mean that the difference is considered statistically significant (*p* < 0.05).

Variables	Case (*n* = 82)	CAR≤0.0396 (*n* = 44)	CAR>0.0396 (*n* = 38)	χ^2^	*p*-Value
Age				1.781	0.182
≤45 years	30	19 (43.2)	11 (28.9)		
>45 years	52	25 (56.8)	27 (71.1)		
FIGO stage				0.142	0.706
IB	62	34 (77.3)	28 (73.7)		
IIA	20	10 (22.7)	10 (26.3)		
Maximum tumor size				0.124	0.725
≤4 cm	64	35 (79.5)	29 (76.3)		
>4 cm	18	9 (20.5)	9 (23.7)		
Pathological type				0.093	0.761
Squamous	46	24 (54.5)	22 (57.9)		
Non-squamous	36	20 (45.5)	16 (42.1)		
Neoadjuvant chemotherapy				0.183	0.669
No	58	32 (72.7)	26 (68.4)		
Yes	24	12 (27.3)	12 (31.6)		
Histological grade				0.03	0.862
G1 + G2	44	24 (54.5)	20 (52.6)		
G3	38	20 (45.5)	18 (47.4)		
Depth of invasion				1.276	0.259
<2/3	53	26 (59.1)	27 (71.1)		
≥2/3	29	18 (40.9)	11 (28.9)		
PLR				0.897	0.343
≤163.41	63	32 (72.7)	31 (81.6)		
>163.41	19	12 (27.3)	7 (18.4)		
PNI				3.831	0.0503
≤50.15	18	6 (13.6)	12 (31.6)		
>50.15	64	38 (86.4)	26 (68.4)		
BMI, kg/m^2^				6.786	**0.009**
<19 or >24	37	14 (31.8)	23 (60.5)		
19–24	45	30 (68.2)	15 (39.5)		

FIGO, Federation of Gynecologists and Obstetricians; Depth of invasion, tumor invasion in mm/cervical wall thickness in mm; CAR, C-reactive protein/albumin ratio; PLR, platelet-lymphocyte ratio; PNI, prognostic nutritional index; BMI, body mass index.

**TABLE 6:** Comparison of clinical characteristics between preoperative PLR^lo^ and PLR^hi^ groups in patients with HPV-negative cervical cancer.

Variables	Case (*n* = 82)	PLR ≤ 163.41 (*n* = 63)	PLR > 163.41 (*n* = 19)	χ^2^	*p*-Value
Age				2.572	0.109
≤45 years	30	26 (41.3)	4 (21.1)		
>45 years	52	37 (58.7)	15 (78.9)		
FIGO stage				0.007	0.935
IB	62	47 (74.6)	15 (78.9)		
IIA	20	16 (25.4)	4 (21.1)		
Maximum tumor size				1.116	0.291
≤4 cm	64	47 (74.6)	17 (89.5)		
>4 cm	18	16 (25.4)	2 (10.5)		
Pathological type				0.121	0.728
Squamous	46	36 (57.1)	10 (52.6)		
Non-squamous	36	27 (42.9)	9 (47.4)		
Neoadjuvant chemotherapy				2.170	0.141
No	58	42 (66.7)	16 (84.2)		
Yes	24	21 (33.3)	3 (15.8)		
Histological grade				0.01	0.918
G1 + G2	44	34 (54.0)	10 (52.6)		
G3	38	29 (46.0)	9 (47.4)		
Depth of invasion				0.155	0.694
<2/3	53	40 (63.5)	13 (68.4)		
≥2/3	29	23 (36.5)	6 (31.6)		
PNI				2.169	0.141
≤50.15	18	11 (17.5)	7 (36.8)		
>50.15	64	52 (82.5)	12 (63.2)		
CAR				0.897	0.343
≤0.0396	44	32 (50.8)	12 (63.2)		
>0.0396	38	31 (49.2)	7 (36.8)		
BMI, kg/m^2^				0.091	0.763
<19 or >24	37	29 (46.0)	8 (42.1)		
19–24	45	34 (54.0)	11 (57.9)		

FIGO, Federation of Gynecologists and Obstetricians; Depth of invasion, tumor invasion in mm/cervical wall thickness in mm; CAR, C-reactive protein/albumin ratio; PLR, platelet-lymphocyte ratio; PNI, prognostic nutritional index; BMI, body mass index.

**TABLE 7:** Comparison of clinical characteristics between preoperative PNI^lo^ and PNI^hi^ groups in patients with HPV-negative cervical cancer.

Variables	Case (*n* = 82)	PNI ≤ 50.15 (*n* = 18)	PNI > 50.15 (*n* = 64)	χ^2^	*p*-Value
Age				0.105	0.746
≤45 years	30	6 (33.3)	24 (37.5)		
>45 years	52	12 (66.7)	40 (62.5)		
FIGO stage				0.005	0.946
IB	62	13 (72.2)	49 (76.6)		
IIA	20	5 (27.8)	15 (23.4)		
Maximum tumor size				0.125	0.724
≤4 cm	64	13 (72.2)	51 (79.7)		
>4 cm	18	5 (27.8)	13 (20.3)		
Pathological type				1.272	0.259
Squamous	46	8 (44.4)	38 (59.4)		
Non-squamous	36	10 (55.6)	26 (40.6)		
Neoadjuvant chemotherapy				0.184	0.668
No	58	12 (66.7)	46 (71.9)		
Yes	24	6 (33.3)	18 (28.1)		
Histological grade				0.787	0.375
G1 + G2	44	8 (44.4)	36 (56.2)		
G3	38	10 (55.6)	28 (43.8)		
Depth of invasion				0.832	0.362
<2/3	53	10 (55.6)	43 (67.2)		
≥2/3	29	8 (44.4)	21 (32.8)		
PLR				2.169	0.141
≤163.41	63	11 (61.1)	52 (81.2)		
>163.41	19	7 (38.9)	12 (18.8)		
CAR				3.831	0.0503
≤0.0396	44	6 (33.3)	38 (59.4)		
>0.0396	38	12 (66.7)	26 (40.6)		
BMI, kg/m^2^				0.004	0.948
<19 or >24	37	8 (44.4)	29 (45.3)		
19–24	45	10 (55.6)	35 (54.7)		

FIGO, Federation of Gynecologists and Obstetricians; Depth of invasion, tumor invasion in mm/cervical wall thickness in mm; CAR, C-reactive protein/albumin ratio; PLR, platelet-lymphocyte ratio; PNI, prognostic nutritional index; BMI, body mass index.

### Prognostic Roles of CAR, PLR, PNI in Patients With HPV-Positive and HPV-Negative Cervical Cancer

**FIGURE 1:**
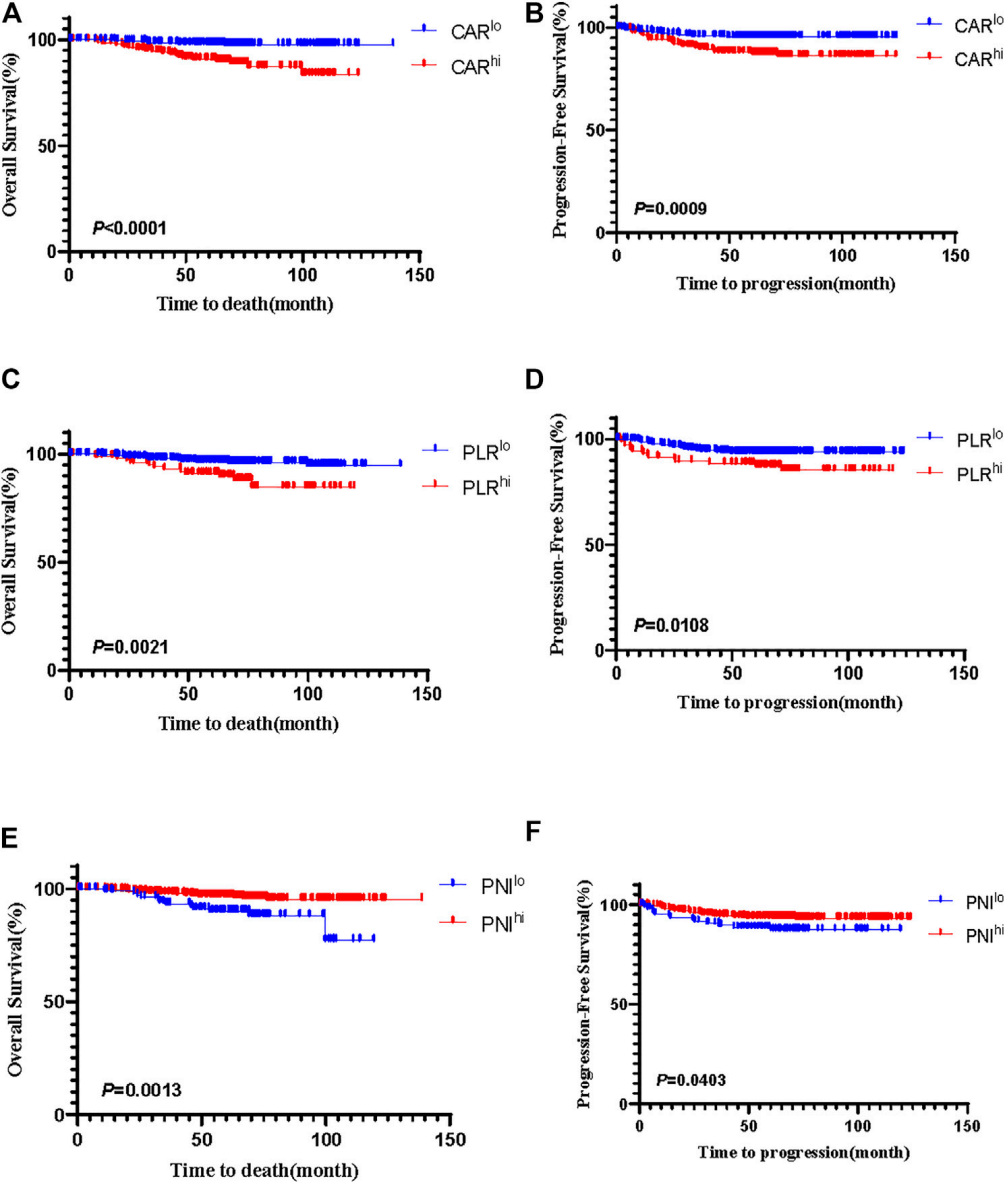
The Kaplan-Meier curves of CAR for OS **(A)** and PFS **(B)**, PLR for OS **(C)** and PFS **(D)**, and PNI for OS **(E)** and PFS **(F)** in patients with HPV-positive cervical cancer.

Kaplan–Meier analyses showed that there was no difference of prognosis between HPV-positive and HPV-negative groups (Supplementary Figure S3). Among 501 patients with HPV-positive cervical cancer, CAR^hi^ (*p* < 0.0001; *p* = 0.0009), PLR^hi^ (*p* = 0.0021; *p* = 0.0108) had worse OS and PFS (Figures 1A–D), while PNI^hi^ (*p* = 0.0013; *p* = 0.0403) had favorable OS and PFS (Figures 1E,F). For the HPV-negative group, none of the three indexes were related to prognosis (all *p* > 0.05) (Supplementary Figure S4).

For HPV-positive group, the multivariate analysis showed that the depth of invasion (HR: 3.651, 95% CI: 1.464–9.103, *p* = 0.005), the levels of PNI (HR: 0.341, 95% CI: 0.156–0.745, *p* = 0.007) and CAR (HR: 5.201, 95% CI: 2.080–13.004, *p* < 0.0001) were independent factors for OS (Table 8). In addition, the depth of invasion (HR: 2.478, 95% CI: 1.218–5.043, *p* = 0.012), the levels of PLR (HR: 1.991, 95% CI: 1.018–3.894, *p* = 0.044) and CAR (HR: 2.769, 95% CI: 1.406–5.455, *p* = 0.003) were independent predictors of PFS (Table 9). For the HPV-negative group, only the depth of invasion (HR: 9.192, 95% CI: 1.016–83.173, *p* = 0.048) was an independent prognostic factor of OS (Table 10), while none of clinicopathological parameters were independent prognostic indicators of PFS (Table 11).

**TABLE 8:** Univariate and multivariate Cox proportional hazards analyses of overall survival in patients with HPV-positive cervical cancer. The bold values mean that the difference is considered statistically significant (*p* < 0.05).

Variables	Univariable analyses HR (95% CI)	*p*-Value	Multivariate analyses HR (95% CI)	*p*-Value
Age		0.642		
≤45 years	1			
>45 years	1.218 (0.529–2.804)			
FIGO stage		0.155		
IB	1			
IIA	1.750 (0.809–3.785)			
Maximum tumor size		0.961		
≤4 cm	1			
>4 cm	0.974 (0.335–2.828)			
Pathological type		0.289		
Squamous	1			
Non-squamous	0.458 (0.108–1.940)			
Neoadjuvant chemotherapy		0.290		
No	1			
Yes	0.522 (0.157–1.741)			
Histological grade		0.412		
G1 + G2	1			
G3	1.403 (0.625–3.150)			
Depth of invasion		**0.004**		**0.005**
<2/3	1		1	
≥2/3	3.820 (1.534–9.514)		3.651 (1.464–9.103)	
PLR		**0.004**		0.125
≤163.41	1			
>163.41	3.152 (1.457–6.818)			
PNI		**0.002**		**0.007**
≤50.15	1		1	
>50.15	0.301 (0.139–0.654)		0.341 (0.156–0.745)	
CAR		**<0.0001**		**<0.0001**
≤0.0396	1		1	
>0.0396	5.620 (2.255–14.003)		5.201 (2.080–13.004)	
BMI, kg/m^2^		0.640		
<19 or >24	1			
19–24	0.832 (0.386–1.796)			

FIGO, Federation of Gynecologists and Obstetricians; Depth of invasion, tumor invasion in mm/cervical wall thickness in mm; CAR, C-reactive protein/albumin ratio; PLR, platelet-lymphocyte ratio; PNI, prognostic nutritional index; BMI, body mass index.

**TABLE 9:** Univariate and multivariate Cox proportional hazards analyses of progression free survival in patients with HPV-positive cervical cancer. The bold values mean that the difference is considered statistically significant (*p* < 0.05).

Variables	Univariable analyses HR (95% CI)	*p*-Value	Multivariate analyses HR (95% CI)	*p*-Value
Age		0.935		
≤45 years	1			
>45 years	0.972 (0.495–1.910)			
FIGO stage		0.392		
IB	1			
IIA	1.332 (0.691–2.568)			
Maximum tumor size		0.386		
≤4 cm	1			
>4 cm	1.414 (0.646–3.094)			
Pathological type		0.739		
Squamous	1			
Non-squamous	0.852 (0.332–2.186)			
Neoadjuvant chemotherapy		0.799		
No	1			
Yes	0.899 (0.395–2.046)			
Histological grade		0.767		
G1 + G2	1			
G3	1.105 (0.573–2.129)			
Depth of invasion		**0.005**		**0.012**
<2/3	1		1	
≥2/3	2.744 (1.356–5.554)		2.478 (1.218–5.043)	
PLR		**0.013**		**0.044**
≤163.41	1		1	
>163.41	2.314 (1.191–4.498)		1.991 (1.018–3.894)	
PNI		**0.044**		0.252
≤50.15	1			
>50.15	0.500 (0.255–0.983)			
CAR		**0.002**		**0.003**
≤0.0396	1		1	
>0.0396	2.976 (1.515–5.846)		2.769 (1.406–5.455)	
BMI, kg/m^2^		0.723		
<19 or >24	1			
19–24	0.890 (0.467–1.696)			

FIGO, Federation of Gynecologists and Obstetricians; Depth of invasion, tumor invasion in mm/cervical wall thickness in mm; CAR, C-reactive protein/albumin ratio; PLR, platelet-lymphocyte ratio; PNI, prognostic nutritional index; BMI, body mass index.

**TABLE 10:** Univariate and multivariate Cox proportional hazards analyses of overall survival in patients with HPV-negative cervical cancer. The bold values mean that the difference is considered statistically significant (*p* < 0.05).

Variables	Univariable analyses HR (95% CI)	*p*-Value	Multivariate analyses HR (95% CI)	*p*-Value
Age		0.312		
≤45 years	1			
>45 years	46.845 (0.027–81020.538)			
FIGO stage		0.312		
IB	1			
IIA	2.541 (0.416–15.517)			
Maximum tumor size		0.893		
≤4 cm	1			
>4 cm	1.167 (0.121–11.223)			
Pathological type		0.111		
Squamous	1			
Non-squamous	6.085 (0.660–56.127)			
Neoadjuvant chemotherapy		0.335		
No	1			
Yes	0.023 (0.000–49.671)			
Histological grade		0.776		
G1 + G2	1			
G3	1.320 (0.212–8.233)			
Depth of invasion		**0.048**		**0.048**
<2/3	1			
≥2/3	9.192 (1.016–83.173)		9.192 (1.016–83.173)	
PLR		0.883		
≤163.41	1			
>163.41	0.848 (0.095–7.591)			
PNI		0.847		
≤50.15	1			
>50.15	0.804 (0.088–7.347)			
CAR		0.464		
≤0.0396	1			
>0.0396	1.959 (0.324–11.850)			
BMI, kg/m^2^		0.225		
<19 or >24	1			
19–24	0.010 (0.000–16.576)			

FIGO, Federation of Gynecologists and Obstetricians; Depth of invasion, tumor invasion in mm/cervical wall thickness in mm; CAR, C-reactive protein/albumin ratio; PLR, platelet-lymphocyte ratio; PNI, prognostic nutritional index; BMI, body mass index.

**TABLE 11:** Univariate and multivariate Cox proportional hazards analyses of progression free survival in patients with HPV-negative cervical cancer.

Variables	Univariable analyses HR (95% CI)	*p*-Value	Multivariate analyses HR (95% CI)	*p*-Value
Age		0.439		
≤45 years	1			
>45 years	2.377 (0.265–21.297)			
FIGO stage		0.293		
IB	1			
IIA	2.680 (0.427–16.812)			
Maximum tumor size		0.515		
≤4 cm	1			
>4 cm	0.034 (0.000–904.981)			
Pathological type		0.147		
Squamous	1			
Non-squamous	5.063 (0.566–45.314)			
Neoadjuvant chemotherapy		0.583		
No	1			
Yes	0.541 (0.060–4.861)			
Histological grade		0.806		
G1 + G2	1			
G3	1.262 (0.196–8.139)			
Depth of invasion		0.218		
<2/3	1			
≥2/3	204.563 (0.043–966070.729)			
PLR		0.477		
≤163.41	1			
>163.41	0.034 (0.000–371.005)			
PNI		0.916		
≤50.15	1			
>50.15	0.887 (0.097–8.121)			
CAR		0.158		
≤0.0396	1			
>0.0396	4.850 (0.542–43.400)			
BMI, kg/m^2^		0.497		
<19 or >24	1			
19–24	0.538 (0.090–3.220)			

FIGO, Federation of Gynecologists and Obstetricians; Depth of invasion, tumor invasion in mm/cervical wall thickness in mm; CAR, C-reactive protein/albumin ratio; PLR, platelet-lymphocyte ratio; PNI, prognostic nutritional index; BMI, body mass index.

All the above data suggested that for patients with HPV-positive FIGO stage IB∼IIA cervical cancer, the depth of invasion, the levels of PNI and CAR are independent predictors of OS, and the depth of invasion, the levels of PLR and CAR are independent predictors of poor PFS. For patients with HPV-negative cervical cancer, none of CAR, PNI and PLR is independent factor of prognosis.

## Discussion

The immune system, which resists pathogenic microorganisms, repairs damage, and maintains stability of internal environment, mainly relies on normal autoimmunity reactions [27]. Some pathogens have been proven to promote tumors, such as cervical cancer (HPV), nasopharyngeal cancer (Epstein-Barr virus), liver cancer (Hepatitis B virus), and gastrointestinal cancer (Helicobacter pylori) [28, 29]. During the occurrence and development of malignant tumors, the inflammatory response plays an essential role [30, 31]. Inflammation is not only involved in all processes of tumor progression, but also affects treatment and prognosis [32, 33]. Crusz et al. believed that immune-cell infiltration of tumors can have a dual role. Both tumor and immune cells of solid tumors secrete tumor necrosis factor-α (TNF-α) which stimulates tumor cell growth survival, invasion, and metastasis, and inflammatory cell trafficking and neoangiogenesis [33]. Besides, tumor-associated macrophages (TAMs) degrade the extracellular matrix (ECM) and basement membrane by releasing a number of proteolytic enzymes, including matrix metalloproteinases (MMPs) and serine proteases, thus facilitating tumor invasion and metastasis [34]. Research has clarified that HPV E6/E7 mRNA decreased the expression of p53 and pRb, increased that of p16, activated the mammalian target of rapamycin (mTOR) signaling pathway, inhibited cell apoptosis, and induced cell proliferation [35, 36]. Studying tumors and the immune system as a whole is the current trend and direction in tumor research.

Previous studies have indicated that inflammation is one of the factors influencing the prognosis of patients with cancer [32, 37]. Our findings suggested that CAR, PLR and PNI levels have predictive effects on the prognosis of patients with HPV-positive early-stage cervical cancer. We noted that among women with FIGO 2018 IB∼IIA cervical cancer, survival was improved in those with lower levels of CAR or PLR, or higher level of PNI. There is an interaction between platelets and tumor cells. Tumor cells stimulate platelet activation through direct contact or the release of platelet-inducing factors, such as adenosine diphosphate (ADP). Platelets activate their surface receptors and secrete a variety of bioactivators that play an important role in tumor cell growth, metastasis, and angiogenesis [38]. An increased platelet counts was identified as an independent prognostic factor of poor survival in lung cancer [39]. CRP is a widely used and important acute-phase serum protein for monitoring infection in the clinic. Although an increased level of CRP might be nonspecific, some studies have found that patients with cancer have a higher CRP level than healthy people [40]. Malnutrition accounts for 20% of all cancer-related deaths [41]. Malnutrition and inflammation suppress the synthesis of serum ALB, which can reflect the nutritional status of patients, as well as the severity, progression, and prognosis of disease [42]. In gastrointestinal cancer, lymphocytes secrete cytokines, such as interferon gamma (IFN-γ) and TNF-α, thereby controlling tumor growth and improving the prognosis. A decline in the number and function of lymphocytes could weaken immunological surveillance and defense [43]. In addition to prognosis, the alteration of systemic inflammation and nutritional status may influence the response to immune checkpoint inhibitors (ICIs). In non-small cell lung cancer (NSCLC), CPR, ALB, PNI and NLR were reported to be associated with the response to ICIs [44–46]. Therefore, based on the above indicators, the balance between the immune status and tumor progression in patients with cancer can be better displayed.

In the present study, we proposed to evaluate the prognostic value of inflammatory indexes based on the patients’ HPV infection status. The same results were not observed in both HPV-positive and HPV-negative groups. Before our analyses, there were many studies that explored the relationships between inflammatory indexes and prognosis in cervical cancer, but none of them paid attention to the HPV infection status of the patients [47, 48]. The above results revealed that in patients with HPV-negative cervical cancer, the inflammatory indexes were not suitable as indicators of prognosis. In addition, our study included cervical cancer patients with stage IB∼IIA according to the FIGO 2018 guidelines, except for those with lymph node metastasis. These results identified low-cost, convenient, and effective preoperative prognostic factors for patients with early-stage cervical cancer.

There are some limitations of this study. First, this study is retrospective. Therefore, there might have been selection bias and analysis bias. However, a large sample size guarantees the sufficient statistical power and reduces the risk of bias in HPV-positive group. In this study, one of the weaknesses is the low number of HPV-negative cases. It is vital to expand the number of HPV-negative group to avoid bias in our future studies. Second, in the present study, there were differences in the pathological types between the HPV-positive and negative groups. Thus, to avoid these problems, it is crucial to carry out prospective researches.

In summary, we found that in patients with HPV-positive cervical cancer, the depth of invasion, the levels of CAR and PNI are independent predictors of OS, and the depth of invasion, the levels of PLR and CAR are independent predictors of PFS. In HPV-negative cases, three inflammation indexes had no prognostic value. Interestingly, when the analysis was limited to patients with negative lymph node metastasis, CAR, PLR and PNI still had significant prognostic value. Our research verified that clarifying the HPV infection status will be more accurate for the application of preoperative inflammatory parameters in the prognosis of cervical cancer.

## Data Availability

The original contributions presented in the study are publicly available. This data can be found here: Research Data Deposit (www.researchdata.org.cn), RDD number RDDA202101939.

## References

[1] Bray F Ferlay J Soerjomataram I Siegel RL Torre LA Jemal A. Global Cancer Statistics 2018: GLOBOCAN Estimates of Incidence and Mortality Worldwide for 36 Cancers in 185 Countries. CA: A Cancer J Clinicians (2018) 68(6):394–424. 10.3322/caac.21492 30207593

[2] Sadri Nahand J Moghoofei M Salmaninejad A Bahmanpour Z Karimzadeh M Nasiri M Pathogenic Role of Exosomes and microRNAs in HPV‐mediated Inflammation and Cervical Cancer: A Review. Int J Cancer (2020) 146(2):305–20. 10.1002/ijc.32688 31566705 PMC6999596

[3] Roden RBS Stern PL. Opportunities and Challenges for Human Papillomavirus Vaccination in Cancer. Nat Rev Cancer (2018) 18(4):240–54. 10.1038/nrc.2018.13 29497146 PMC6454884

[4] Zhao F Qiao Y. Cervical Cancer Prevention in China: a Key to Cancer Control. The Lancet (2019) 393(10175):969–70. 10.1016/s0140-6736(18)32849-6 30860036

[5] Cohen PA Jhingran A Oaknin A Denny L. Cervical Cancer. The Lancet (2019) 393(10167):169–82. 10.1016/s0140-6736(18)32470-x 30638582

[6] Bhatla N Aoki D Sharma DN Sankaranarayanan R. Cancer of the Cervix Uteri. Int J Gynecol Obstet (2018) 143(Suppl. 2):22–36. 10.1002/ijgo.12611 30306584

[7] Namikawa T Shimizu S Yokota K Tanioka N Munekage M Uemura S Neutrophil-to-lymphocyte Ratio and C-Reactive Protein-To-Albumin Ratio as Prognostic Factors for Unresectable Advanced or Recurrent Gastric Cancer. Langenbecks Arch Surg (2021). 10.1007/s00423-021-02356-w 34652563

[8] Yang Y Gao P Song Y Sun J Chen X Zhao J The Prognostic Nutritional index Is a Predictive Indicator of Prognosis and Postoperative Complications in Gastric Cancer: A Meta-Analysis. Eur J Surg Oncol (Ejso) (2016) 42(8):1176–82. 10.1016/j.ejso.2016.05.029 27293109

[9] McMillan DC Canna K McArdle CS. Systemic Inflammatory Response Predicts Survival Following Curative Resection of Colorectal Cancer. Br J Surg (2003) 90(2):215–9. 10.1002/bjs.4038 12555298

[10] Ayhan A Günakan E Alyazıcı İ Haberal N Altundağ Ö Dursun P. The Preoperative Albumin Level Is an Independent Prognostic Factor for Optimally Debulked Epithelial Ovarian Cancer. Arch Gynecol Obstet (2017) 296(5):989–95. 10.1007/s00404-017-4511-9 28875365

[11] Zhang F Liu Z Liang J Liu S Wu K Zhang F Association between Preoperative Serum Albumin and Prognosis in Patients with Adrenocortical Carcinoma after Primary Resection: a Retrospective Study. BMC Cancer (2021) 21(1):961. 10.1186/s12885-021-08689-5 34445989 PMC8393459

[12] Zhou T Zhan J Hong S Hu Z Fang W Qin T Ratio of C-Reactive Protein/Albumin Is an Inflammatory Prognostic Score for Predicting Overall Survival of Patients with Small-Cell Lung Cancer. Sci Rep (2015) 5:10481. 10.1038/srep10481 26084991 PMC4471724

[13] Deng Y Zhao Y Qin J Huang X Wu R Zhou C Prognostic Value of the C-Reactive Protein/Albumin Ratio and Systemic Immune-Inflammation Index for Patients with Colorectal Liver Metastasis Undergoing Curative Resection. Pathol Oncol Res (2021) 27:633480. 10.3389/pore.2021.633480 34257601 PMC8262228

[14] Stevens L Pathak S Nunes QM Pandanaboyana S Macutkiewicz C Smart N Prognostic Significance of Pre-operative C-Reactive Protein and the Neutrophil-Lymphocyte Ratio in Resectable Pancreatic Cancer: a Systematic Review. HPB (2015) 17(4):285–91. 10.1111/hpb.12355 25431369 PMC4368390

[15] Kinoshita A Onoda H Imai N Iwaku A Oishi M Tanaka K The C-Reactive Protein/albumin Ratio, a Novel Inflammation-Based Prognostic Score, Predicts Outcomes in Patients with Hepatocellular Carcinoma. Ann Surg Oncol (2015) 22(3):803–10. 10.1245/s10434-014-4048-0 25190127

[16] Smith RA Bosonnet L Raraty M Sutton R Neoptolemos JP Campbell F Preoperative Platelet-Lymphocyte Ratio Is an Independent Significant Prognostic Marker in Resected Pancreatic Ductal Adenocarcinoma. Am J Surg (2009) 197(4):466–72. 10.1016/j.amjsurg.2007.12.057 18639229

[17] Davis AN Afshar-Kharghan V Sood AK. Platelet Effects on Ovarian Cancer. Semin Oncol (2014) 41(3):378–84. 10.1053/j.seminoncol.2014.04.004 25023353 PMC4100073

[18] Zhang Y Wang L Liu Y Wang S Shang P Gao Y Preoperative Neutrophil-Lymphocyte Ratio before Platelet-Lymphocyte Ratio Predicts Clinical Outcome in Patients with Cervical Cancer Treated with Initial Radical Surgery. Int J Gynecol Cancer (2014) 24(7):1319–25. 10.1097/IGC.0000000000000219 25033256

[19] Asher V Lee J Innamaa A Bali A. Preoperative Platelet Lymphocyte Ratio as an Independent Prognostic Marker in Ovarian Cancer. Clin Transl Oncol (2011) 13(7):499–503. 10.1007/s12094-011-0687-9 21775277

[20] Chan AWH Chan SL Wong GLH Wong VWS Chong CCN Lai PBS Prognostic Nutritional Index (PNI) Predicts Tumor Recurrence of Very Early/Early Stage Hepatocellular Carcinoma after Surgical Resection. Ann Surg Oncol (2015) 22(13):4138–48. 10.1245/s10434-015-4516-1 25801356

[21] Migita K Takayama T Saeki K Matsumoto S Wakatsuki K Enomoto K The Prognostic Nutritional index Predicts Long-Term Outcomes of Gastric Cancer Patients Independent of Tumor Stage. Ann Surg Oncol (2013) 20(8):2647–54. 10.1245/s10434-013-2926-5 23463091

[22] Kanda M Fujii T Kodera Y Nagai S Takeda S Nakao A. Nutritional Predictors of Postoperative Outcome in Pancreatic Cancer. Br J Surg (2010) 98(2):268–74. 10.1002/bjs.7305 20960457

[23] Wang W-J Li Y Zhu J Gao M-j. Shi J-p. Huang Y-q. Prognostic Values of Systemic Inflammation Response (SIR) Parameters in Resectable Cervical Cancer. Dose-Response (2019) 17(1):155932581982954. 10.1177/1559325819829543 PMC6393952 30833874

[24] Han X Liu S Yang G Hosseinifard H Imani S Yang L Prognostic Value of Systemic Hemato-Immunological Indices in Uterine Cervical Cancer: A Systemic Review, Meta-Analysis, and Meta-Regression of Observational Studies. Gynecol Oncol (2021) 160(1):351–60. 10.1016/j.ygyno.2020.10.011 33092868

[25] Nishijima TF Muss HB Shachar SS Tamura K Takamatsu Y. Prognostic Value of Lymphocyte-To-Monocyte Ratio in Patients with Solid Tumors: A Systematic Review and Meta-Analysis. Cancer Treat Rev (2015) 41(10):971–8. 10.1016/j.ctrv.2015.10.003 26481060

[26] National Comprehensive Cancer Network. Clinical Practice Guidelines in Oncology. Cervical Cancer. Version 1. 2022 Available At: https://www.nccn.org/professionals/physician_gls/pdf/cervical.pdf (Accessed October 26, 2021).

[27] Yurkovetskiy LA Pickard JM Chervonsky AV. Microbiota and Autoimmunity: Exploring New Avenues. Cell Host & Microbe (2015) 17(5):548–52. 10.1016/j.chom.2015.04.010 25974297 PMC4535992

[28] Chen Y-P Chan ATC Le Q-T Blanchard P Sun Y Ma J. Nasopharyngeal Carcinoma. The Lancet (2019) 394(10192):64–80. 10.1016/s0140-6736(19)30956-0 31178151

[29] Amieva M Peek RM Jr. Pathobiology of helicobacter Pylori-Induced Gastric Cancer. Gastroenterology (2016) 150(1):64–78. 10.1053/j.gastro.2015.09.004 26385073 PMC4691563

[30] de Visser KE Eichten A Coussens LM. Paradoxical Roles of the Immune System during Cancer Development. Nat Rev Cancer (2006) 6(1):24–37. 10.1038/nrc1782 16397525

[31] Janssen LME Ramsay EE Logsdon CD Overwijk WW. The Immune System in Cancer Metastasis: Friend or Foe? J Immunotherapy Cancer (2017) 5(1):79. 10.1186/s40425-017-0283-9 PMC5644253 29037250

[32] Diakos CI Charles KA McMillan DC Clarke SJ. Cancer-related Inflammation and Treatment Effectiveness. Lancet Oncol (2014) 15(11):e493–e503. 10.1016/s1470-2045(14)70263-3 25281468

[33] Crusz SM Balkwill FR. Inflammation and Cancer: Advances and New Agents. Nat Rev Clin Oncol (2015) 12(10):584–96. 10.1038/nrclinonc.2015.105 26122183

[34] Lin Y Xu J Lan H. Tumor-associated Macrophages in Tumor Metastasis: Biological Roles and Clinical Therapeutic Applications. J Hematol Oncol (2019) 12(1):76. 10.1186/s13045-019-0760-3 31300030 PMC6626377

[35] Hoppe-Seyler K Bossler F Braun JA Herrmann AL Hoppe-Seyler F. The HPV E6/E7 Oncogenes: Key Factors for Viral Carcinogenesis and Therapeutic Targets. Trends Microbiol (2018) 26(2):158–68. 10.1016/j.tim.2017.07.007 28823569

[36] Zhou J Peng C Li B Wang F Zhou C Hong D Transcriptional Gene Silencing of HPV16 E6/E7 Induces Growth Inhibition via Apoptosis *In Vitro* and *In Vivo* . Gynecol Oncol (2012) 124(2):296–302. 10.1016/j.ygyno.2011.10.028 22056554

[37] Nie D Gong H Mao X Li Z. Systemic Immune-Inflammation index Predicts Prognosis in Patients with Epithelial Ovarian Cancer: A Retrospective Study. Gynecol Oncol (2019) 152(2):259–64. 10.1016/j.ygyno.2018.11.034 30558974

[38] Schlesinger M. Role of Platelets and Platelet Receptors in Cancer Metastasis. J Hematol Oncol (2018) 11(1):125. 10.1186/s13045-018-0669-2 30305116 PMC6180572

[39] Maráz A Furák J Varga Z Kahán Z Tiszlavicz L Hideghéty K. Thrombocytosis Has a Negative Prognostic Value in Lung Cancer. Anticancer Res (2013) 33(4):1725–30. 23564823

[40] Siemes C Visser LE Coebergh J-WW Splinter TAW Witteman JCM Uitterlinden AG C-reactive Protein Levels, Variation in the C-Reactive Protein Gene, and Cancer Risk: the Rotterdam Study. J Clin Oncol (2006) 24(33):5216–22. 10.1200/JCO.2006.07.1381 17114654

[41] Obermair A Simunovic M Isenring L Janda M. Nutrition Interventions in Patients with Gynecological Cancers Requiring Surgery. Gynecol Oncol (2017) 145(1):192–9. 10.1016/j.ygyno.2017.01.028 28173966

[42] Gupta D Lis CG. Pretreatment Serum Albumin as a Predictor of Cancer Survival: A Systematic Review of the Epidemiological Literature. Nutr J (2010) 9:69. 10.1186/1475-2891-9-69 21176210 PMC3019132

[43] Ferrone C Dranoff G. Dual Roles for Immunity in Gastrointestinal Cancers. J Clin Oncol (2010) 28(26):4045–51. 10.1200/JCO.2010.27.9992 20644090 PMC4872327

[44] Duchemann B Remon J Naigeon M Mezquita L Ferrara R Cassard L Integrating Circulating Biomarkers in the Immune Checkpoint Inhibitor Treatment in Lung Cancer. Cancers (2020) 12(12):3625. 10.3390/cancers12123625 PMC7761725 33287347

[45] Shoji F Takeoka H Kozuma Y Toyokawa G Yamazaki K Ichiki M Pretreatment Prognostic Nutritional index as a Novel Biomarker in Non-small Cell Lung Cancer Patients Treated with Immune Checkpoint Inhibitors. Lung Cancer (2019) 136:45–51. 10.1016/j.lungcan.2019.08.006 31437663

[46] Oya Y Yoshida T Kuroda H Mikubo M Kondo C Shimizu J Predictive Clinical Parameters for the Response of Nivolumab in Pretreated Advanced Non-small-cell Lung Cancer. Oncotarget (2017) 8(61):103117–28. 10.18632/oncotarget.21602 29262550 PMC5732716

[47] Huang H Liu Q Zhu L Zhang Y Lu X Wu Y Prognostic Value of Preoperative Systemic Immune-Inflammation index in Patients with Cervical Cancer. Sci Rep (2019) 9(1):3284. 10.1038/s41598-019-39150-0 30824727 PMC6397230

[48] Zhang W Liu K Ye B Liang W Ren Y. Pretreatment C-Reactive Protein/albumin Ratio Is Associated with Poor Survival in Patients with Stage IB-IIA Cervical Cancer. Cancer Med (2018) 7(1):105–13. 10.1002/cam4.1270 29193777 PMC5773960

