# Supplementary material for: Pretreatment C-Reactive Protein/Albumin Ratio is Associated With Poor Survival in Patients With 2018 FIGO Stage IB-IIA HPV-Positive Cervical Cancer
Source: Pathol Oncol Res. 2021 Dec 21;27:1609946. doi: 10.3389/pore.2021.1609946 (PMC8724028; doi:10.3389/pore.2021.1609946)

Supplementary Figures

Supplementary Fig 1. OS **(A)** and PFS **(B)** of cervical cancer patients according to the postoperative adjuvant therapy.

(A)

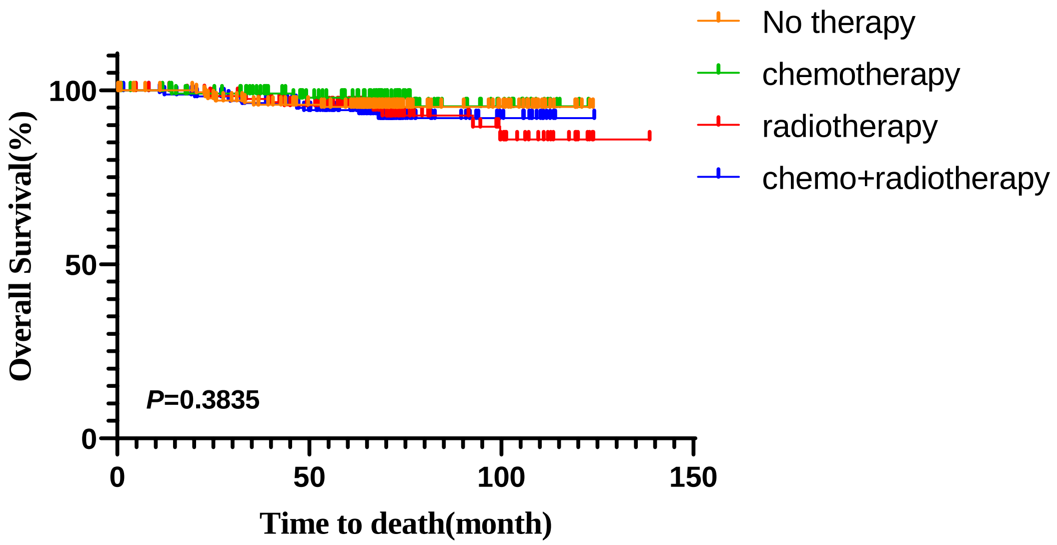

(B)

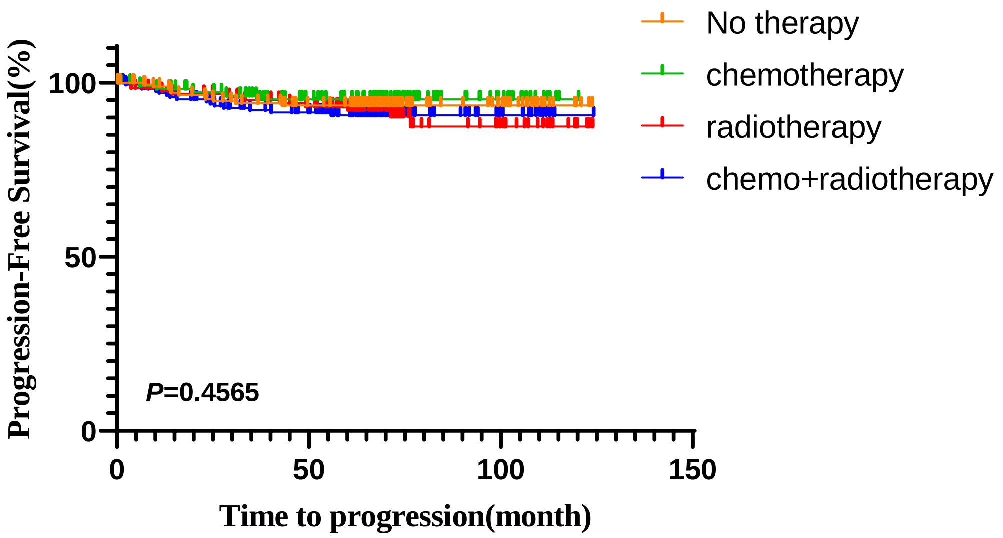

Supplementary Fig 2. ROC curves of CAR, PLR and PNI in cervical cancer patients.

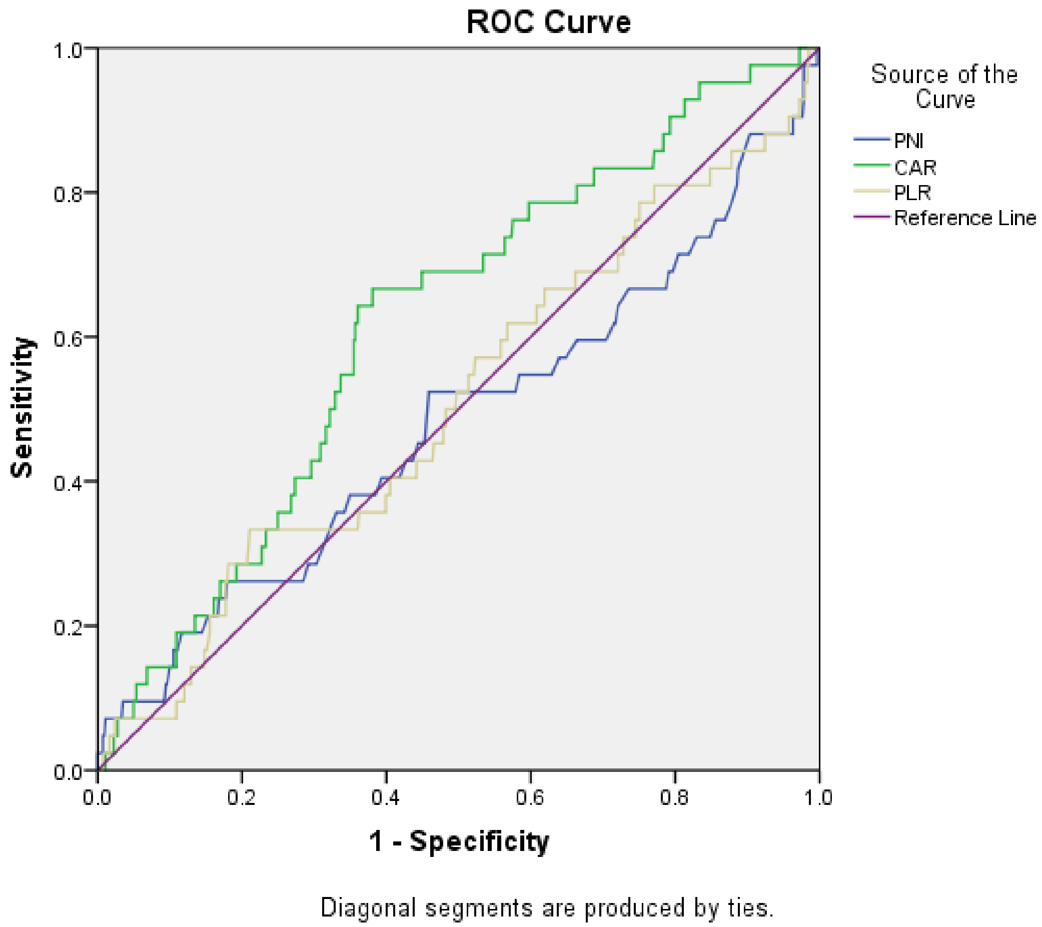

Supplementary Fig 3. OS **(A)** and PFS **(B)** of cervical cancer patients according to the HPV infection status.

(A)

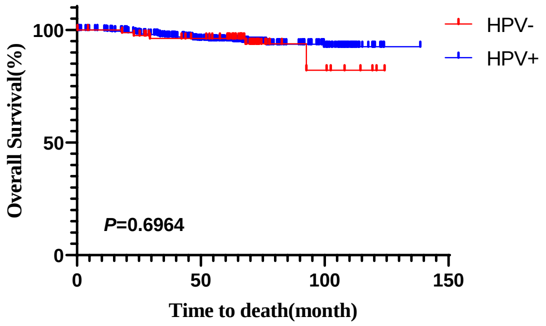

(B)

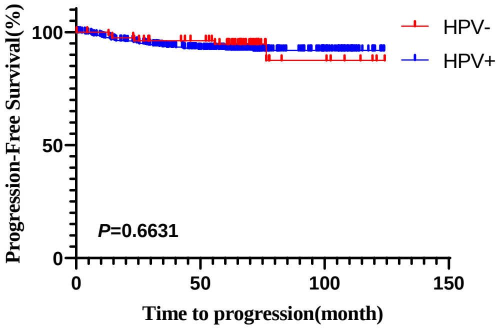

Supplementary Fig 4. The Kaplan-Meier curves of CAR for OS **(A)** and PFS **(B)**, PLR for OS **(C)** and PFS **(D)**, and PNI for OS **(E)** and PFS **(F)** in patients with HPV-negative cervical cancer.

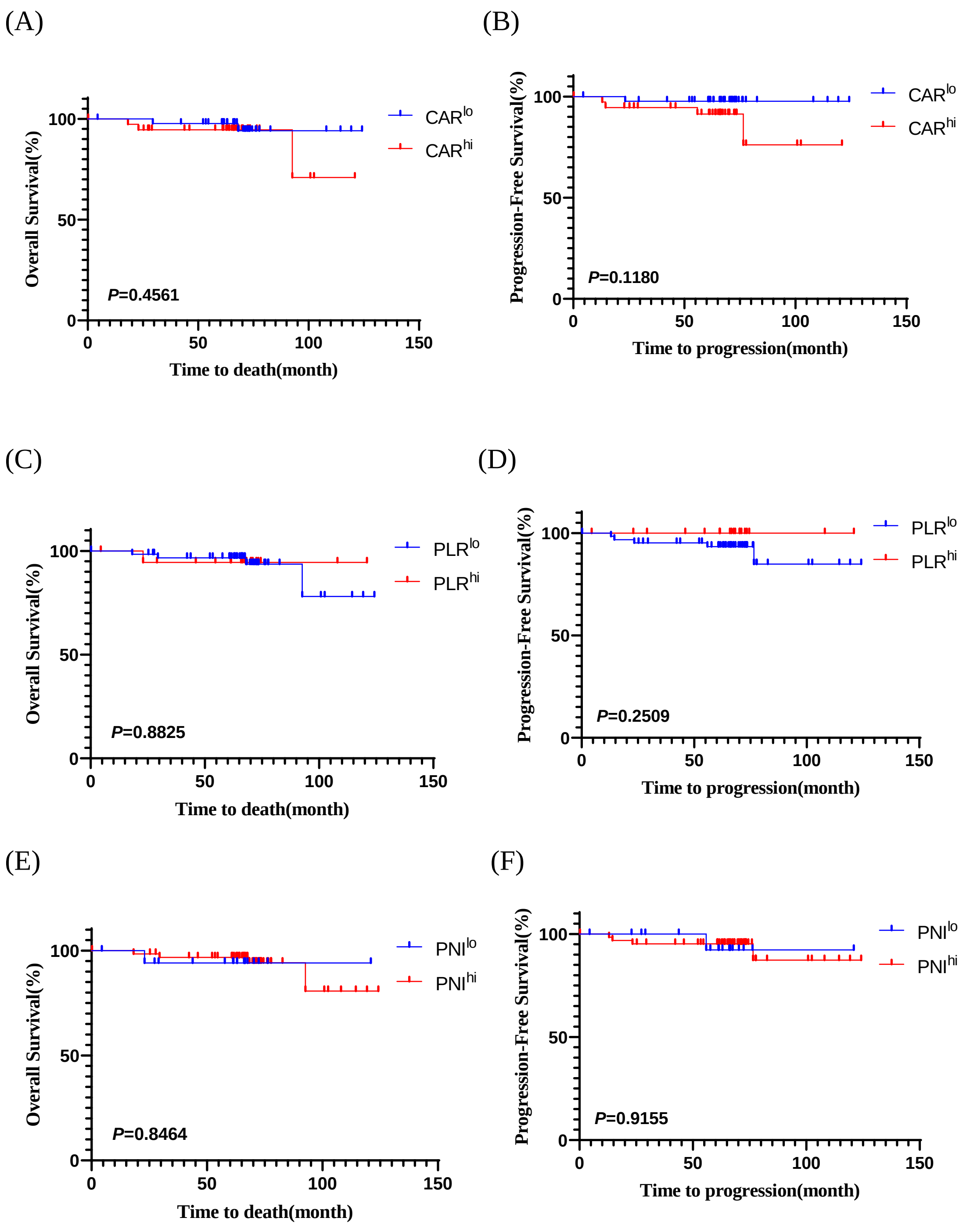

Supplement: Supplementary file 1 [file DataSheet1.pdf]
